# Characterization of the *Pinus massoniana* Transcriptional Response to *Bursaphelenchus xylophilus* Infection Using Suppression Subtractive Hybridization

**DOI:** 10.3390/ijms140611356

**Published:** 2013-05-28

**Authors:** Liang Xu, Zhen-Yu Liu, Kai Zhang, Quan Lu, Jun Liang, Xing-Yao Zhang

**Affiliations:** 1 College of Forestry, Beijing Forestry University, Beijing 100083, China; E-Mail: xuliang19824@163.com; 2 Research Institute of Forest Ecology, Environment and Protection, Chinese Academy of Forestry, Beijing 100091, China; E-Mails: zk_shuibing@yahoo.com.cn (K.Z.); luquan@caf.ac.cn (Q.L.); liangjun@caf.ac.cn (J.L.); 3 College of Plant Protection, Shandong Agricultural University, Tai’an 271018, Shandong, China

**Keywords:** pine wilt disease, differentially expressed genes, suppression subtractive hybridization, *Pinus massoniana*

## Abstract

Pine wilt disease (PWD) caused by pine wood nematode (PWN), *Bursaphelenchus xylophilus*, is the most destructive diseases of pine and poses a threat of serious economic losses worldwide. Although several of the mechanisms involved in disease progression have been discovered, the molecular response of *Pinus massoniana* to PWN infection has not been explored. We constructed four subtractive suppression hybridization cDNA libraries by taking time-course samples from PWN-inoculated Masson pine trees. One-hundred forty-four significantly differentially expressed sequence tags (ESTs) were identified, and 124 high-quality sequences with transcriptional features were selected for gene ontology (GO) and individual gene analyses. There were marked differences in the types of transcripts, as well as in the timing and levels of transcript expression in the pine trees following PWN inoculation. Genes involved in signal transduction, transcription and translation and secondary metabolism were highly expressed after 24 h and 72 h, while stress response genes were highly expressed only after 72 h. Certain transcripts responding to PWN infection were discriminative; pathogenesis and cell wall-related genes were more abundant, while detoxification or redox process-related genes were less abundant. This study provides new insights into the molecular mechanisms that control the biochemical and physiological responses of pine trees to PWN infection, particularly during the initial stage of infection.

## 1. Introduction

Pine wilt disease (PWD) is caused by the pine wood nematode (PWN), *Bursaphelenchus xylophilus*, which is believed to be native to North America. Previously, PWD only damaged exotic pine trees in North America, but it has recently spread to Asian and European countries, including Japan, China, South Korea and Portugal, and PWN was recently reported in Spain [1–3]. PWD is the most destructive pine disease, causing significant economic losses around the world, especially in Asia. PWD kills 1,000,000 m^3^ of pine trees annually in Japan [4] and damaged approximately 7811 ha of pines in Korea by 2005 [5]. In China, economic losses due to PWD were estimated to be 2.5 billion Chinese RMB (approximately 400 million USD) [6]. *Pinus* spp. are the main hosts of *B. xylophilus*, and *Monochamus* spp. beetles are a major vector, feeding on multiple pine trees and introducing the nematodes into the trees through feeding wounds [7]. Once the pine trees are infected, they are usually killed rapidly, and no effective treatment measures are available. Despite many advances on the study of PWD, the pathogenic mechanism of PWD has not been clearly defined.

PWD symptoms are thought to develop in distinct early and advanced stages. During the early stage, typical symptoms are observed near the inoculation site, such as necrosis and destruction of the cortex, phloem tissue and cambium; the destruction of cortex resin canals; the formation of wound periderm in the cortex parenchyma around the resin canals; and ethylene release [8]. During the advanced stage, ethylene production, cambium damage and xylem embolisms increase, ultimately resulting in wilting needles and the death of the tree [8–10].

Accompanying these symptoms, an innate hypersensitive reaction (HR) occurs, resulting in the release of phenolics, the synthesis of toxins and phytoalexins and the compartmentalization of xylem and other tissues, followed by the flooding of the tracheid with oleoresin and toxic substances [9]. Further studies have shown that the HR is activated by a genetic program, wherein resistance genes recognize certain effectors and initiate a resistance response that is frequently linked to rapid cell death [11].

Certain high throughput screening procedures, including suppression subtractive hybridization (SSH) and cDNA microarray technologies, have been used to identify differentially expressed genes in the plant-nematode interaction system [5,12,13]. In particular, comparative transcriptomic analyses with confirmation by Illumina deep sequencing have introduced a novel technique for the study of plant response to nematode infection [14,15]. Recent studies have revealed many molecular genetic events associated with PWD progression using such high throughput screening procedures. Shin *et al.* [5] isolated and analyzed upregulated or newly induced genes in PWN-inoculated Japanese red pine (*P. densiflora*) using an annealing control primer system and SSH. Significant changes in abundance were found for gene transcripts related to defense, secondary metabolism and transcription. As the disease progressed, other gene transcripts encoding pathogenesis-related proteins were expressed. In addition, pinosylvin synthases and metallothioneins were also more abundant in PWN-inoculated trees than in non-inoculated trees [5]. Santos *et al.* [12] identified expressed sequence tags (ESTs) in *P. pinaster* and *P. pinea* that were inoculated with PWN using the SSH technique and found that a tree defense response occurred at the molecular level at the initial stage of the disease; this response involved mainly the oxidative stress response, the production of lignin and ethylene and the posttranscriptional regulation of nucleic acids. Hirao *et al.* [13] constructed seven SSH *P. thunbergii* cDNA libraries from trees after inoculation with PWN and discovered that the expression levels of antimicrobial peptide and putative pathogenesis-related genes (e.g., PR-1b, 2, 3, 4, 5, 6) were much higher in susceptible trees at every time point, whereas the expression levels of PR-9, PR-10 and cell wall-related genes (e.g., hydroxyproline-rich glycoprotein precursor and extensin) were higher in resistant trees. The physiological changes that occur as PWD progresses have been characterized anatomically and biochemically, but the genetic events, such as changes in transcript profiles that may be associated with physiological changes, are poorly understood.

Masson pine (*P. massoniana*) is one of the most extensively distributed pine species in China. Most of these trees are found in pure forests. Unfortunately, these forests have been severely damaged by PWD because of Masson pine’s high susceptibility to PWD. The disease has spread during the last three decades and has caused serious damage to this particular species [6]. However, the response of the Masson pine to PWN infection at the molecular level has not been elucidated. In this study, we constructed four SSH libraries from Masson pines at two stages, 24 h and 72 h post-PWD inoculation. Control inoculations were performed with water to compare Masson pine gene expression patterns in response to PWN infection. The genes that were regulated by PWN inoculation were identified by comparing libraries, and the interesting results from the 24 h and 72 h treatments were further studied with real-time quantitative reverse-transcription polymerase chain reaction (qRT-PCR) analysis.

## 2. Results

### 2.1. Analysis of cDNA Libraries Constructed by SSH

To identify the *P. massoniana* genes that were differentially expressed during the early response stages of PWN inoculation, four SSH libraries were constructed using mRNAs from PWN-inoculated and water-inoculated stems that were sampled at 24 h and 72 h after inoculation (PM-24h and PM-72h, respectively). From each library, 384 clones were selected randomly for dot-blot hybridization. A total of 665 dots (339 dots for the two PM-24h SSH libraries and 326 dots for the two PM-72h SSH libraries) were identified as significantly differentially expressed genes between PWN-inoculated and water-inoculated *P. massoniana* based on a normalized PWN-inoculation/water-inoculation signal intensity ratio with more than 2.0 or less than 0.5 (Table S1). A total of 144 spots, including 61 dots for the two PM-24h SSH libraries and 83 dots for the two PM-72h SSH libraries, were sequenced. After removing the low-quality region, vector and adaptor sequences, 124 high-quality sequences of the 144 sequences from the four libraries were selected for further analysis (Table S1). A total of five contigs and 32 singletons were present in the two PM-24h SSH libraries and the contigs comprised 23 ESTs, with a redundancy of 42%. The two PM-72h SSH libraries contained 49 unique ESTs, including four contigs and 45 singletons, with 35% redundancy. The insert length varied from 132 to 1051 bp, and the median length ranged from 428 to 459 bp, depending on the library. Summaries of the library data are shown in Table 1. The EST sequences have been deposited in the NCBI dbEST database (accession numbers JZ349248 to JZ349371).

### 2.2. Functional Classification and Identification of differentially Expressed Genes in PWN-Inoculated *P. massoniana* Trees

**Figure 1:**
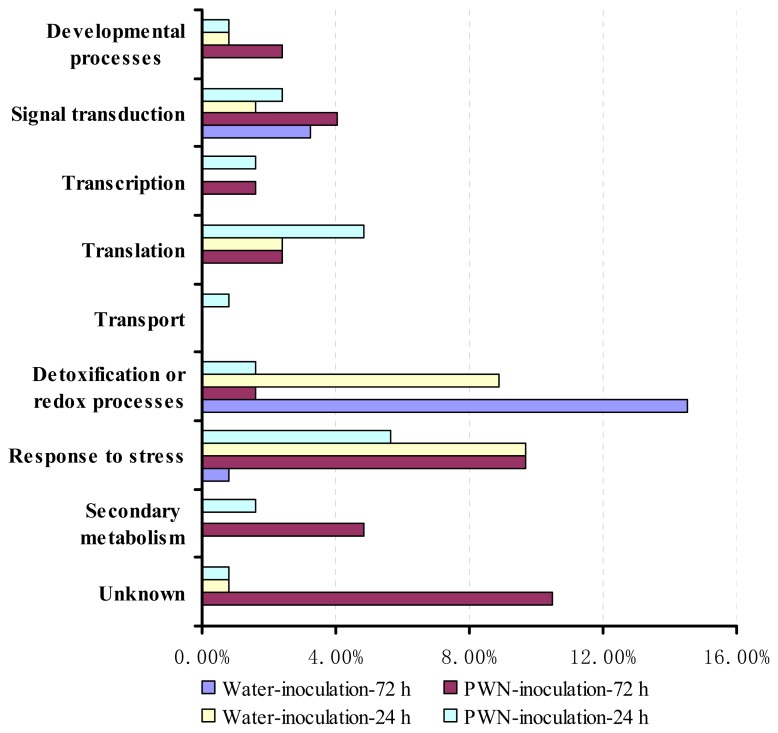
Functional classification of the ESTs from the SSH libraries. For library construction, the mRNAs from pine wood nematode (PWN)-inoculated *Pinus massoniana* stems that were sampled at 24 h and 72 h after inoculation were used as the tester set, and the mRNAs from water-inoculated stems were used as the driver set.

We classified and analyzed the genes from the four SSH libraries according to the gene ontology (GO) classes of their closest orthologues in *Arabidopsis*. These genes were involved in developmental processes, signal transduction, transcription and translation, transport, detoxification or redox processes and responses to stress and secondary metabolism after inoculation (Figure 1). However, genes involved in signal transduction, transcription and translation and secondary metabolism were highly expressed after 24 h and 72 h, while genes involved in transport were highly expressed only after 24 h. During PWD development, stress response genes were highly expressed only after 72 h; in addition, genes involved in developmental processes and several unknown genes were also highly expressed only after 72 h. In addition to the GO analysis, we also performed individual gene analyses to examine the relationships of the differentially expressed genes to the biochemical and physiological responses of trees during the early stages after PWN inoculation. The most noteworthy of these genes are shown in Table 2 and are reviewed in the Discussion section.

Fifty-two upregulated genes were identified in *P. massoniana* trees at 24 h and 72 h after PWN inoculation. Twenty genes were identified as upregulated at only 24 h after inoculation. Of these genes, six were involved stress responses, including genes encoding dirigent-like protein, heat-shock protein (HSP) 90 and PR proteins, such as thaumatin-like family protein (PR-5), chitin recognition protein (PR-4) and chitinase (PR-3). Two upregulated genes were involved in detoxification or redox processes: glutathione peroxidase and 2-alkenal reductase. One upregulated gene, FAD/NAD(P)-binding oxidoreductase family protein, was involved in secondary metabolism. Twenty-nine genes were upregulated only at 72 h after inoculation. Of these genes, 11 were involved in stress responses: mannose/glucose-specific lectin, HSP 70, AAA-type ATPase family protein, cell-wall protein (lp5), BCL-2-associated athanogene 6, ubiquitin family protein, ubiquitin conjugating-like enzyme, lipid transfer protein (PR-14), adenine nucleotide alpha hydrolase-like superfamily protein, RPS 2 protein and pentatricopeptide (PPR) repeat-containing protein. Two upregulated genes were involved in detoxification or redox processes: glyoxalase 1 family protein and Cyt_b561_FRRS1-like-containing protein. Five upregulated genes were involved in secondary metabolism: cinnamyl alcohol dehydrogenase 5 (CAD), chalcone and stilbene synthase family protein (CHS), caffeic acid *O*-methyltransferase (COMT), caffeoyl-CoA *O*-methyltransferase (CCoAOMT) and phenylcoumaran benzylic ether reductase (PCBER). Genes encoding the jasmonate ZIM domain-containing protein PMXY 121, PMXY 205, the phosphate-responsive protein, PMXY 130, PMXY 210, and methionine synthase 2 PMXY 149, PMXY 166 were upregulated at both 24 h and 72 h after inoculation (Table 2).

In contrast, 15 genes were identified as downregulated in PWN-inoculated *P. massoniana* trees 24 h and 72 h after inoculation. Eleven genes exhibited decreased expression at only 24 h after inoculation. Of these genes, five were involved in stress responses: HSP 18.2, HSP 101, calcium binding protein lp8, drought stress responsive protein and high mobility group B3. Three genes exhibited decreased expression only at 72 h after inoculation. Of these genes, one gene encoding an aluminum-induced protein with YGL and LRDR motifs was classified as a stress response gene and one gene encoding a heavy metal-associated domain-containing protein was classified as a detoxification or redox process gene. Genes encoding metallothionein 3 Contig 02 and Contig 04 were downregulated at both 24 h and 72 h after PWN inoculation (Table 2).

### 2.3. Validation of Differential Expression using Selected SSH Clones and qRT-PCR

To confirm the reliability of the SSH results, four genes were selected and analyzed by qRT-PCR to evaluate the changes in their expression in response to PWN inoculation. These genes were either upregulated or downregulated in PWN-inoculated *P. massoniana* trees at 24 h and 72 h after inoculation. One selected downregulated gene was HSP 18.2 (Contig 06), which was involved in stress responses at 24 h after inoculation. The three selected upregulated genes were upregulated only at 72 h: no pollen germination 1 (NPG1) (PMXY 165), which is involved in developmental processes and CAD (PMXY 152) and CHS (PMXY 187), both of which are involved in secondary metabolism.

**Figure 2:**
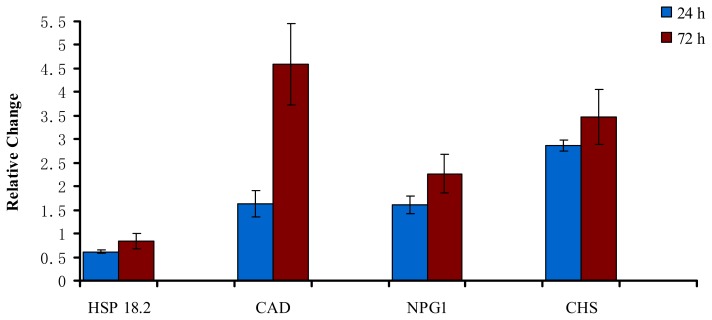
Quantitative real-time PCR of transcripts that were differentially expressed at 24 h and 72 h from PWN-inoculated Masson pine trees. The change is expressed as the relative difference in gene expression between the PWN-inoculated sample and the water-inoculated sample.

The qRT-PCR data confirmed that NPG1, CAD and CHS were upregulated in PWN-inoculated trees, and that the expression levels were gene-specific, ranging from 1.60 to 4.54 (Figure 2). The three genes were upregulated at both 24 h and 72 h, and the expression levels of the three genes were much higher at 72 h than at 24 h (Figure 2). Although the expression levels of HSP 18.2 were higher at 72 h than at 24 h, this gene was downregulated both at 24 h and at 72 h in PWN-inoculated *P. massoniana* trees. Overall, the results of the qRT-PCR analysis were consistent with the SSH analysis.

## 3. Discussion

To examine the gene expression profiles of *P. massoniana* in response to PWN inoculation, we constructed four SSH libraries from PWN- and water-inoculated *P. massoniana* stems sampled at 24 h and 72 h after inoculation. Overall, GO analysis showed that genes involved in signal transduction, transcription, translation and secondary metabolism were more highly expressed in PWN-inoculated trees than in water-inoculated trees at both 24 h and 72 h, and genes involved in stress responses were only more highly expressed in the PWN-inoculated trees at 72 h. However, genes that were involved in detoxification or redox processes were expressed at lower levels in PWN-inoculated trees than in the water-inoculated trees at both 24 h and 72 h (Figure 1). These results suggest that genes involved in responses to stress and secondary metabolism were expressed in the early stage of the *P. massoniana* response to PWN infection.

Genes encoding PR proteins were found to be highly expressed in *P. massoniana* after PWN inoculation, including PR-3 (chitinase, PMXY 146), PR-4 (chitin recognition protein, PMXY 128), PR-5 (thaumatin-like protein, Contig 05, PMXY 127) and PR-14 (lipid transfer protein, PMXY 158) (Figure 1, Table 2). PR proteins are expressed by host plants in response to pathological stimuli, and they normally accumulate not only locally, at the site of inoculation, but also systemically following exposure to biotic and abiotic stress factors [16]. PRs have been confirmed to be involved in pine and PWN interactions. Studies have revealed that PR gene expression was induced more quickly and to a higher level in the early stages of pine responses to PWN infection [5,13,17]. PR-2 (β-1,3-glucanase-like protein), PR-3 and PR-4 are thought to be involved in degrading the cell walls of fungal pathogens from the *Ceratocystis* genus, which are known to infect pine trees concomitantly with PWN [18]. PR-5 proteins exhibit antifungal activity by binding to (1,3)-β-d-glucans [19,20]. In this study, PR-3, PR-4 and PR-5 were found to be upregulated in the PM-24h library, indicating the response of *P. massoniana* to PWN infection. In addition, PR-14 (lipid transfer protein) produces a direct cytotoxic effect on fungal cells that is mediated by membrane permeabilization and inhibits fungal growth [21]. The gene that encodes PR-14 was found to be upregulated in *P. pinaster* at 3 h and 24 h after PWN inoculation [12,22] and was also found to be upregulated in *P. massoniana* 72 h after PWD infestation in our study.

Mannose/glucose-specific lectin and HSP 70, which are associated with stress responses, were also upregulated in *P. massoniana* at 72 h after PWD infection (Figure 1, Table 2). Plant lectins are entomotoxic proteins that are present in many species, and they participate in a general defense against a multitude of plant pathogens, including nematodes [23]. Ricin B-related lectin expression was upregulated in PWN-infected *P. pinea* [22]. HSP 70 is required for the folding of nascent proteins and intracellular transportation in addition to stress responses [24]. In the interaction between soybean plants and soybean cyst nematodes, Klink *et al.* [25] observed the induction of HSP 70 and reactive oxygen species (ROS) responsive genes, such as lipoxygenase and superoxidase dismutase, characteristic in response to soybean cyst nematode infection, suggesting that HSP 70 may contribute to maintaining a properly functioning environment for other defense responses.

Many genes that are involved in detoxification and redox processes were identified by the present study. During PWN invasion, metabolites released by the action of enzymes in nematode saliva, such as cellulase [26], as well as the host’s own secondary metabolites, which are normally restricted to specialized cells or subcellular compartments, can trigger the exposure of plant cells to highly toxic oxygen species [27]. We identified a number of genes that were involved in oxidative stress, including those encoding metallothionein 3 (Contig 02 downregulated in PM-24h and Contig 04 downregulated in PM-72h), a heavy metal-associated domain-containing protein (Contig 03 downregulated in PM-72h), GPX (PMXY 134 upregulated in PM-24h), 2-alkenal reductase (PMXY 136 upregulated in PM-24h), glyoxalase 1 family protein (PMXY 168 upregulated in PM-72h) and Cyt_b561_ FRRS1-like-containing protein (PMXY 170 upregulated in PM-72h) (Figure 1, Table 2).

A novel NADPH-dependent oxidoreductase 2-alkenal reductase gene was upregulated in the PM-24h library in this study. This protein can catalyze the reduction of reactive carbonyls that are produced by biomembrane lipid peroxidation in PWN-inoculated pine trees [28–32]. In addition, an aldo/keto reductase, an NADPH-dependent oxidoreductase that is involved in the detoxification of reactive carbonyls [33], was shown to be induced at 24 h after PWD-infection in *P. pinaster* [22]. The 2-alkenal reductase appears to play an important role in the antioxidative defense during the early stages following PWN inoculation.

The GPX gene is induced in maritime pine seedlings in response to water deficits and can catalyze the oxidation of glutathione in the presence of H_2_O_2_ to yield oxidized glutathione and water [34]. A GPX gene was found to be upregulated in the PM-24h library in our study. A cytochrome b561 gene was also upregulated in the PM-72h library. Cytochrome b561 is believed to catalyze the reduction of monodehydroascorbate (MDHA), resulting in the generation of a fully reduced ascorbate molecule [35]. These two molecules of reduced ascorbate can be used to reduce H_2_O_2_ to water by l-ascorbate peroxidase (APX), with the concomitant regeneration of two molecules of MDHA [36]. The APX gene has been verified to respond to PWN infection in PWN-inoculated Japanese red pine [5]. Therefore, the accumulation of GPX and Cyt_b561_FRRS1-like-containing protein provides evidence of H_2_O_2_ increases during the early stages following PWN inoculation in *P. massoniana* trees.

Metallothioneins are involved in metal homeostasis and heavy metal detoxification, and they are highly expressed in tissues under intense oxidative stress [37]. In the Norway spruce (*Picea abies*), metallothioneins accumulate in the needles under ozone stress and have been implicated in the maintenance of intracellular redox potential via the detoxification of ROS [38]. However, in our study, genes encoding metallothionein 3 (Contig 02 in PM-24 h and Contig 04 in PM-72 h) and a heavy metal-associated domain-containing protein (Contig 03 in PM-72 h) were identified as downregulated in the early stages of PWD progression in *P. massoniana* (Table 2). Hirao *et al.* [13] also reported that an EST that putatively encodes a metallothionein-like protein was significantly downregulated in susceptible *P. thunbergii* trees at 1 and 3 d. The response of such genes in *P. massoniana* and *P. thunbergii* suggests that they are likely involved in susceptible pine responses to PWN.

Genes involved in detoxification or redox processes were found to be induced at both 24 h and 72 h in PWN-inoculated *P. massoniana* trees in this study (Figure 1, Table 2). Shin *et al.* [5] found that genes related to oxidative stress were strongly induced or upregulated by PWN infection, implying that the inoculated trees were under severe oxidative stress during the nematode infection process. However, the upregulation of oxidative stress-related proteins is believed to play important roles in the maintenance of intracellular redox balance and in stress response/tolerance. Thus, the observed oxidative stress-related responses indicated that ROS accumulation and defense signaling may have been induced by 24 h or 72 h in the PWN-infected trees. Hirao *et al.* [13] proposed that the rapid induction of defense response genes, such as PR protein-encoding genes, is induced by oxidative stress-related gene expression during the early stage of PWN infection, and our study provides evidence from *P. massoniana* to support this hypothesis.

Secondary metabolites are important defense agents in conifers [5,13]. In this study, many genes involved in secondary metabolism responded to PWN inoculation, including the genes encoding methionine synthase 2, CAD, PCBER, COMT, CCoAOMT, CHS and other enzymes (Table 2). These proteins are specifically responsible for the production of secondary metabolites related to the phenylpropanoid pathway, flavonoid/stilbenoid pathway and the lignin biosynthetic pathway. Anatomical studies of PWN infection have demonstrated the accumulation of lignin and suberin-like substances around the resin canals in the cortex [39,40]. In our study, five genes encoding the lignin pathway components, methionine synthase 2, CAD, PCBER, COMT and CCoAOMT, were upregulated at 72 h after PWN inoculation. In addition, a dirigent-like protein gene was also upregulated at 24 h after PWD inoculation. The dirigent-like protein can mediate stereospecific lignin precursor couplings in association with H_2_O_2_ and may be involved in cell wall modification/lignification [41,42]. Cell wall-mediated resistance is the first line of plant defense against pathogens. Therefore, these findings indicate that the activation of resistant genes is associated with lignin synthesis. The upregulation of cell wall-related genes contributes to the strength of cell walls, providing a very effective defense against PWN infection; these events may restrict PWN migration. Despite these findings, the role of these genes in secondary metabolism in relation to PWN infection remains unclear [13,43,44].

Transcriptional regulators are key factors in the expression of specific genes, and they ensure cellular responses to internal and external stimuli [45,46]. In response to PWN inoculation, significant changes in the expression of genes associated with defense, detoxification or redox processes and secondary metabolism were observed, implying that these genes are transcriptionally regulated. Therefore, we analyzed the expression levels of several transcription factors in the four libraries. Genes encoding putative PHD finger family proteins and zinc finger proteins (C_2_H_2_ type) were found to be upregulated in the PM-24h library and genes encoding C_3_HC_4_-type RING finger proteins, and a putative La domain-containing protein were found to be upregulated in the PM-72h library. Thus, these regulators were differentially expressed in PWN-inoculated *P. massoniana* trees and could control the biochemical and physiological responses of *P. massoniana* to PWN infection. The transcription factors that were associated with PWN infection, and their regulation patterns in relation to downstream genes should be studied further.

## 4. Experimental Section

### 4.1. Plant Material and Nematode Culture

Non-resistant (non-selected) 50 cm tall three-year-old *P. massoniana* trees, which were obtained from the Laoshan Forest Farm of Chun’an County in Zhejiang, China, were potted at the Research Institute of Subtropical Forestry in Zhejiang, China in June 2008. The *P. massoniana* trees were maintained in an environmental growth chamber with a relative humidity of 80%, a photoperiod of 16 h and temperatures of 26–30 °C for three months before inoculation. The PWN isolate *B. xylophilus* BXY03 was originally isolated from a diseased *P. massoniana* tree and confirmed as virulent using an inoculation test. The *B. xylophilus* BXY03 was then reared on fungal hyphae of *Botrytis cinerea*, which were grown on potato dextrose agar (PDA) medium at 25 °C for 7 days. Prior to inoculation, the PWNs were re-isolated from the medium using the Baermann funnel method [47].

### 4.2. PWN Inoculation and Sampling Time

*P. massoniana* trees were inoculated as described by Futai and Furuno [48]. In brief, a suspension of 1500 nematodes was pipetted into a small longitudinal wound (3–5 cm) made with a scalpel on the main stem, approximately 10 cm above the soil level. The inoculated wounds were covered with Parafilm to prevent the inoculum from drying. Sterile water (without nematodes) was applied to similarly injured *P. massoniana* trees as mock-infected samples. Stem tissues from 5 cm below the inoculated stem site were collected from the inoculated and mock-infected samples at 24 h and 72 h after inoculation. A 2 cm segment (from 5 cm to 7 cm below the inoculated stem site) of each stem was cut, frozen immediately in liquid nitrogen and stored at −80 °C for further analysis.

### 4.3. RNA Extraction and cDNA Synthesis

Saplings of three-year-old *P. massoniana* were used for inoculation with *B. xylophilus* BXY03. One longitudinal wound (approximately 3–5 mm) on each stem at 10 cm high of the sapling was made using a sterilized scalpel, and a suspension of 1500 nematodes was pipetted into each wound. Then, the inoculated wounds were sealed with parafilm to prevent desiccation and contamination. Stems inoculated with sterile water were used as control. Stem tissues with 2 cm length, from 5 cm to 7 cm below the inoculation site, were collected from each stem at 24 h and 72 h after inoculation and frozen immediately in liquid nitrogen for further RNA extraction (Figure S1). Total RNA was isolated from the stem samples using the RNeasy Plant Mini Kit (QIAGEN, Hilden, Germany) by following the protocol. The integrity and purity of the isolated RNA were evaluated using 1.0% agarose gel electrophoresis and spectrophotometry with GeneQuant (GeneQuant II, Pharmacia Biotech, Piscataway, NJ, USA). cDNAs were synthesized using a Super SMART PCR cDNA Synthesis Kit (Clontech, Mountain View, CA, USA) according to the manufacturer’s instructions.

### 4.4. Construction of SSH Libraries and Dot-Blot Hybridization

SSH libraries were constructed using a PCR-Select cDNA Subtraction Kit (Clontech, Mountain View, CA, USA). Four subtractive libraries were constructed from samples taken from PWN and water-inoculated *P. massoniana* trees at 24 h and 72 h after inoculation, including forward (*i.e.*, PWN-inoculated treatment as the tester and the water-inoculated control as the driver) and reverse (*i.e.*, water-inoculated treatment as the tester and the PWN-inoculated control as the driver) subtracted libraries. The SSH cDNA libraries were constructed according to the manufacturer’s instructions. In brief, tester and driver cDNAs were digested with *Rsa*I and ligated to adaptors and two hybridizations and two PCR amplifications were performed to enrich the differentially expressed sequences. The secondary PCR products were purified and inserted into a pMD-18-T Easy Vector (TaKaRa, Otsu, Shiga, Japan), which was then transformed into *Escherichia coli* DH5a competent cells. Blue/white selection was conducted with Luria-Bertani (LB) agar plates containing ampicillin, isopropyl-d-thiogalactopyranoside (IPTG) and X-gal. A set of 384 white clones was randomly selected from each SSH cDNA library and differentially expressed cDNA fragments were amplified by nested PCR with nested primer 1 (5′-TCGAGCGGCCGCCCGGAGGT-3′) and nested primer 2 (5′-AGCGTG GTCGCGGCCGAGGT-3′). Following amplification, 0.6 μL of each PCR product from the two SSH libraries from samples taken at the same times were spotted onto a nylon membrane (Millipore, St. Charles, MO, USA) and pre-hybridized at 68 °C for 2 to 3 h. Two types of probes were generated from the 2nd round of subtracted PCR products, which were produced using the same method that was used to construct the SSH libraries; the samples were digested with *Rsa*I and labelled with P^32^. The two types of probes were then hybridized with two of the same pre-hybridized nylon membranes at 68 °C for 14 h, followed by washing and incubation with a chromogenic reagent. A ScanArray 4000 Standard Biochip Scanning System and its QuantArray software (Packard Biochip Technologies, Inc: Billerica, MA, USA) were applied for scanning and data capturing. Differentially expressed genes were selected randomly based on a normalized PWN-inoculation/water-inoculation signal intensity ratio with more than 2.0 or less than 0.5.

### 4.5. DNA Sequencing and Data Analysis

The clones that were identified by dot-blot hybridization were sequenced using an automated ABI 3730 DNA sequencing system (Applied Biosystems, Foster City, CA, USA). Vector masking and trimming, as well as adaptor removal, were performed with the online VecScreen tool [49]. Contaminating bacterial, mitochondrial and ribosomal RNA genes were identified by a BLASTN search and were removed along with sequences of <100 bases and *E* > 1. The DNA sequence assembly program CAP3 [50] was used to assemble the ESTs into contigs with 75% minimum homology and a 30 base minimum overlap. The BLASTX algorithm was used to perform similarity searches of the individual and clustered ESTs against the GenBank non-redundant *Arabidopsis* databases. Gene ontology (GO) annotation analysis [51] was used for the functional assignment of matched genes.

### 4.6. Quantitative Real-Time PCR (qRT-PCR)

To verify that the library gene results reflected true differential expression, qRT-PCR was performed using the total RNA that were initially isolated for SSH library construction. Gene-specific primers for 18S rRNA, HSP 18.2 (Contig 06), CAD (PMXY 152), NPG1 (PMXY 165) and CHS (PMXY 187) were designed with Primer Express 2.0 (Table 3). After reverse transcription with AMV reverse transcriptase (TaKaRa, Otsu, Shiga, Japan), the reaction product was diluted 100-fold with sterile water. Primer specificity was confirmed by standard PCR and electrophoresis in a 2% agarose gel, and the reaction efficiency was evaluated relative to standard curves generated from four samples from a 10 × dilution series. Real-time qPCR was performed in an Applied Biosystems ABI 7500 Real-Time System with SYBR Green. For each reaction of 20 μL, 2 μL of diluted cDNA was used. The Standard Cycling Program described in the user manual was used for amplification. Melting curve profiles were analyzed to ensure single product amplification, and the PCR products were reconfirmed by electrophoresis in a 2% agarose gel to verify the generation of a single product of the expected size. Each sample was tested in triplicate. Transcript abundance was normalized to that of 18S rRNA using the ΔΔ*C*t method, as described previously [52], and the data for each gene in the PWN-inoculated samples were compared with the data from water-inoculated samples.

## 5. Conclusions

Pine wilt disease, caused by *B. xylophilus*, is the most destructive pine disease. The host plant, *P. massoniana*, is extensively distributed across China, and it is highly susceptible to severe damage by PWD. To assess the pine molecular genetics associated with PWD, *i.e.*, the transcriptional changes in *P. massoniana* trees following PWN infection, we constructed four subtractive suppression hybridization (SSH) cDNA libraries using time-course sampling of PWN-inoculated Masson pine trees. The genes that exhibited differential expression in response to PWN inoculation were identified by comparing these different libraries. Markedly different transcript profiles were found following PWN inoculation. These genes were mainly related to defense, secondary metabolism and transcription. Certain transcripts were differentially regulated in response to infection; pathogenesis-related genes and cell wall-related genes were upregulated, while detoxification or redox process-related genes were downregulated. The expression levels of genes of interest were compared between 24 h and 72 h and further analyzed by qRT-PCR to confirm differential gene expression.

Because the molecular response of the Masson pine to PWN infection was previously unknown, these results provided novel and fundamental information about the molecular response of the Masson pine to PWN infection and provide new insights into the molecular mechanisms underlying the biochemical and physiological responses of pine trees after PWN infection, particularly at the initial stage following infection.

## Supplementary Information



## Figures and Tables

**Table 1:** General characteristics of the subtractive suppression hybridization (SSH) libraries.

	PM-24 h	PM-72 h
Library and EST summary		
Number of cDNAs sequenced	61	83
Mean read length 1	458.5	427.6
Number of high-quality ESTs	55	69

Clustering results		
Number of assembled ESTs 2	23	24
Number of contigs (A)	5	4
Number of singletons (B)	32	45
Number of assembled sequences (A + B)	37	49

Contig sizes		
2–4 ESTs 3	3	2
5–7 ESTs	1	1
8–13 ESTs	1	0
>14 ESTs	0	1

1 Mean EST length after vector masking and end trimming;

2 The EST assembly parameters were 75% minimum match with a 30-base minimum overlap;

3 ESTs, expressed sequence tags.

**Table 2:** Characterization of genes selected from subtractive suppression hybridization (SSH) libraries constructed with mRNA from PWN-inoculated *Pinus massoniana* stems sampled at 24 h and 72 h after PWN inoculation. The signal ratio indicates the normalized PWN-inoculation/water-inoculation ratio.

PM-24h					PM-72h				
	
Clone ID	BLASTX	*E* Value	Signal intensity ratio	Clustered EST No.	Clone ID	BLASTX	*E* Value	Signal intensity ratio	Clustered EST No.
**Developmental process**									

PMXY 148	Pectin methylesterase 6 *Arabidopsis* AT4G02330.1	6 × 10^−84^	2.07 (Upregulated)		PMXY 165	No pollen germination 1 *Arabidopsis* AT2G43040.1	1 × 10^−7^	2.06 (Upregulated)	
	
PMXY 120	Polygalacturonase *Arabidopsis* AT1G80170.1	4 × 10^−18^	0.10 (Downregulated)		PMXY 155	Acyl carrier protein 4 *Arabidopsis* AT4G25050.1	9 × 10^−28^	2.30 (Upregulated)	
	
					PMXY 186	Aldehyde dehydrogenase 11A3 *Arabidopsis* AT2G24270.4	2 × 10^−15^	2.02 (Upregulated)	

**Signal transduction**									

PMXY 121	Jasmonate ZIM domain-containing *Arabidopsis* AT1G74950.1	2 × 10^−3^	2.14 (Upregulated)		Contig 01	Calcium-binding protein *Arabidopsis* AT2G46600.1	2 × 10^−25^	0.11 (Downregulated)	4
	
PMXY 131	Calreticulin 1a *Arabidopsis* AT1G56340.2	1 × 10^−123^	2.10 (Upregulated)		PMXY 153	Calcium-binding protein *Arabidopsis* AT2G46600.1	3 × 10^−12^	2.48 (Upregulated)	
	
PMXY 130	Phosphate-responsive protein *Arabidopsis* AT4G08950.1	1 × 10^−36^	2.10 (Upregulated)		PMXY 205	Jasmonate ZIM domain-containing *Arabidopsis* AT1G19180.2	8 × 10^−3^	2.47 (Upregulated)	
	
PMXY 099	Phosphoesterase family protein *Arabidopsis* AT2G26870.1	4 × 10^−28^	0.08 (Downregulated)		PMXY 160	Response regulator 3 *Arabidopsis* AT1G59940.1	1 × 10^−35^	2.01 (Upregulated)	
	
PMXY 108	Phosphatidylinositol *n*-acetylglucosaminyltransferase subunit p *Arabidopsis* AT2G39445.1	2 × 10^−37^	0.12 (Downregulated)		PMXY 167	RAB GTPase homolog A2D *Arabidopsis* AT5G59150.1	3 × 10^−29^	2.19 (Upregulated)	
	
					PMXY 210	Phosphate-responsive protein *Arabidopsis* AT4G08950.1	2 × 10^−40^	2.05 (Up-regulated)	

**Transcription**									

PMXY 151	PHD finger family protein *Arabidopsis* AT2G02470.2	3 × 10^−48^	2.03 (Upregulated)		PMXY 173	C_3_HC_4_-type RING finger protein *Arabidopsis* AT5G22920.1	4 × 10^−20^	2.15 (Upregulated)	
	
PMXY 147	Zinc finger protein (C_2_H_2_ type) *Arabidopsis* AT2G28200.1	1 × 10^−3^	2.43 (Upregulated)		PMXY 192	La domain-containing protein *Arabidopsis* AT4G32720.2	8 × 10^−17^	2.30 (Upregulated)	

**Translation**									

PMXY 139	60S ribosomal protein L10 *Arabidopsis* AT1G26910.1	2 × 10^−38^	2.17 (Upregulated)		PMXY 204	60S ribosomal protein L38 *Arabidopsis* AT3G59540.1	2 × 10^−8^	2.65 (Upregulated)	
	
PMXY 126	60S ribosomal protein L24 *Arabidopsis* AT2G36620.1	1 × 10^−51^	2.33 (Upregulated)		PMXY 212	Serine-rich proteins *Arabidopsis* AT3G56500.1	8 × 10^−7^	2.15 (Upregulated)	
	
PMXY 141	60S ribosomal protein L29 *Arabidopsis* AT5G02610.1	5 × 10^−54^	2.08 (Upregulated)		PMXY 185	GTP-binding Elongation factor Tu family protein *Arabidopsis* AT5G60390.3	6 × 10^−86^	2.04 (Upregulated)	
	
PMXY 142	60S ribosomal protein L27a-2 *Arabidopsis* AT1G23290.1	1 × 10^−51^	2.37 (Upregulated)						
	
PMXY 133	40S ribosomal protein S11-2 *Arabidopsis* AT3G48930.1	1 × 10^−41^	2.23 (Upregulated)						
	
PMXY 140	Translation initiation factor eIF-4A *Arabidopsis* AT3G13920.4	1 × 10^−142^	2.50 (Upregulated)						
	
PMXY 098	RNA-binding protein Nova-1-like *Arabidopsis* AT5G04430.1	5 × 10^−9^	0.09 (Down-regulated)						
	
PMXY 089	RNA recognition motif (RRM)-containing protein *Arabidopsis* AT1G14340.1	3 × 10^−5^	0.08 (Down-regulated)						
	
PMXY 107	60S ribosomal protein L15 *Arabidopsis* AT4G16720.1	2 × 10^−7^	0.05 (Down-regulated)						

**Transport**									

PMXY 124	TOM1-like protein 2-like isoform 2 *Arabidopsis* AT4G32760.2	4 × 10^−24^	2.31 (Upregulated)						

**Detoxification or redox processes**									

Contig 02	Metallothionein 3 *Arabidopsis* AT3G15353.1	6 × 10^−9^	0.04 (Downregulated)	11	Contig 03	Heavy-metal-associated domaincontaining protein *Arabidopsis* AT1G01490.2	1 × 10^−6^	0.10 (Downregulated)	3
	
PMXY 134	Glutathione peroxidase *Arabidopsis* AT4G11600.1	2 × 10^−71^	2.48 (Upregulated)		Contig 04	Metallothionein 3 *Arabidopsis* AT3G15353.1	6 × 10^−9^	0.09 (Downregulated) 15	
	
PMXY 136	2-alkenal reductase *Arabidopsis* AT5G16970	3 × 10^−23^	2.05 (Upregulated)		PMXY 168	Glyoxalase 1 family protein *Arabidopsis* AT1G15380.2	3 × 10^−12^	2.29 (Upregulated)	
	
					PMXY 170	Cyt_b561_FRRS1-likecontaining protein *Picea sitchensis* ADM73751.1	2 × 10^−4^	2.04 (Upregulated)	

**Response to stress**									

Contig 05	Thaumatin-like family protein *Arabidopsis* AT4G11650.1	4 × 10^−15^	2.55 (Upregulated)	2	Contig 09	Mannose/glucose-specific lectin *Arabidopsis* AT1G19715.3	8 × 10^−14^	1.73 (Upregulated)	2
	
Contig 06	Heat shock protein 18.2 *Arabidopsis* AT5G59720.1	6 × 10^−42^	0.10 (Downregulated)	2	PMXY 172	Heat shock protein 70 *Arabidopsis* AT5G02500.1	8 × 10^−59^	2.06 (Upregulated)	
	
Contig 07	Calcium-binding protein lp8 *Arabidopsis* AT1G24620.1	9 × 10^−20^	0.04 (Downregulated)	5	PMXY 190	AAA-type ATPase family protein *Arabidopsis* AT4G02480.1	2 × 10^−21^	2.21 (Upregulated)	
	
Contig 08	Drought stress responsive protein *Pinus pinaster* AJ309123.1	6 × 10^−97^	0.10 (Downregulated)	3	PMXY 189	Cell-wall protein (lp5) *Pinus sylvestris* EU394126.1	2 × 10^−4^	2.42 (Upregulated)	
	
PMXY 127	Thaumatin-like family protein *Arabidopsis* AT4G11650.1	3 × 10^−16^	5.60 (Upregulated)		PMXY 156	BCL-2-associated athanogene 6 *Arabidopsis* AT2G46240.1	3 × 10^−6^	2.14 (Upregulated)	
	
PMXY 135	Dirigent-like protein *Arabidopsis* AT4G11190.1	0.77	2.39 (Upregulated)		PMXY 195	Ubiquitin family protein *Arabidopsis* AT4G02890.4	1 × 10^−111^	2.19 (Upregulated)	
	
PMXY 128	Chitin recognition protein *Arabidopsis* AT3G04720.1	1 × 10^−15^	8.41 (Upregulated)		PMXY 196	Ubiquitin-like conjugating enzyme *Arabidopsis* AT1G64230.5	6 × 10^−52^	2.23 (Upregulated)	
	
PMXY 146	Chitinase *Arabidopsis* AT2G43590.1	2 × 10^−20^	2.45 (Upregulated)		PMXY 158	Lipid transfer protein *Arabidopsis* AT5G59310.1	1 × 10^−11^	2.47 (Upregulated)	
	
PMXY 145	Heat shock protein 90 *Arabidopsis* AT5G52640.1	1 × 10^−65^	2.33 (Upregulated)		PMXY 209	Adenine nucleotide alpha hydrolase-like superfamily protein *Arabidopsis* AT5G14680.1	6 × 10^−43^	2.11 (Upregulated)	
	
PMXY 103	High mobility group B3 *Arabidopsis* AT1G20696.3	1 × 10^−31^	0.03 (Downregulated)		PMXY 201	RPS 2 protein *Arabidopsis* AT4G26090.1	6 × 10^−6^	2.04 (Upregulated)	
	
PMXY 088	Heat shock protein 101 *Arabidopsis* AT1G74310.1	6 × 10^−10^	0.11 (Downregulated)		PMXY 181	Pentatricopeptide (PPR) repeatcontaining protein *Arabidopsis* AT2G22070.1	3 × 10^−22^	2.35 (Upregulated)	
	
					PMXY 214	Aluminum-induced protein with YGL and LRDR motifs *Arabidopsis* AT5G19140.1	1 × 10^−33^	0.14 (Downregulated)	

**Secondary metabolism**									

PMXY 149	Methionine synthase 2 *Arabidopsis* AT3G03780.3	1 × 10^−120^	2.28 (Upregulated)		PMXY 166	Methionine synthase 2 *Arabidopsis* AT3G03780.3	3 × 10^−59^	2.52 (Upregulated)	
	
PMXY 129	FAD/NAD(P)-binding oxidoreductase family protein *Arabidopsis* AT2G35660.1	1 × 10^−22^	2.11 (Upregulated)		PMXY 202	Caffeic acid Omethyltransferase *Pinus taeda* AAC49708.1	2 × 10^−12^	2.08 (Upregulated)	
	
					PMXY 152	Cinnamyl alcohol dehydrogenase 5 *Arabidopsis* AT4G34230.1	9 × 10^−48^	2.42 (Upregulated)	
	
					PMXY 187	Chalcone and stilbene synthase family protein *Arabidopsis* AT5G13930.1	1 × 10^−35^	2.17 (Upregulated)	
	
					PMXY 211	Caffeoyl-CoA O-methyltransferase *Pinus taeda* AF036095.1	3 × 10^−76^	2.19 (Upregulated)	
	
					PMXY 183	Phenylcoumaran benzylic ether reductase *Pinus taeda* AF242490.2	1 × 10^−94^	2.26 (Upregulated)	

**Table 3:** Primer sequences for quantitative real-time PCR.

Candidate gene	Primer sequence
Heat-shock protein 18.2 (HSP 18.2)	5′-TCATACCGCGTGAGAGGTCAA-3′ 5′-AAGGCGAGCATGGAAAACG-3′
Cinnamyl alcohol dehydrogenase (CAD)	5′-AGCATGGAGGAAGCACAGGAA-3′ 5′-TCCATGGCCGTGTTGATGTAG-3′
No pollen germination 1 (NPG1)	5′-TTGGAGCTGTATCATGCAGCC-3′ 5′-CACCAGTTGACCAATGGAAGC-3′
Chalcone and stilbene synthase family protein (CHS)	5′-GGATCCAGATTCAACTTCGCC-3′ 5′-TGGTTGAGGCATTCCAGCA-3′
18S rRNA	5′-CGGCTACCACATCCAAGGAA-3′ 5′-GCTGGAATTACCGCGGCT-3′
