# Supplementary material for: Characterization of the Pinus massoniana Transcriptional Response to Bursaphelenchus xylophilus Infection Using Suppression Subtractive Hybridization
Source: Int J Mol Sci. 2013 May 28;14(6):11356–75. doi: 10.3390/ijms140611356 (PMC3709736; doi:10.3390/ijms140611356)
Supplement: Supplementary file 1 [file ijms-14-11356-s001.pdf]

Supplementary Information

Figure S1. Inoculation of three-year-old *Pinus massoniana* saplings with *Bursaphelenchus xylophilus* and sample collection for RNA extraction. One longitudinal wound (approximately 3–5 mm) on each stem at 10 cm high of the sapling was made using sterilized scalpel, and a suspension of 1500 nematodes was pipetted into each wound. Then, the inoculated wounds were sealed with Parafilm to prevent desiccation and contamination. Stems inoculated with sterile water were used as control. Stem tissues with 2 cm length, from 5 cm to 7 cm below the inoculation site, was collected from each stem at 24 h and 72 h after inoculation, and frozen immediately in liquid nitrogen for further RNA extraction.

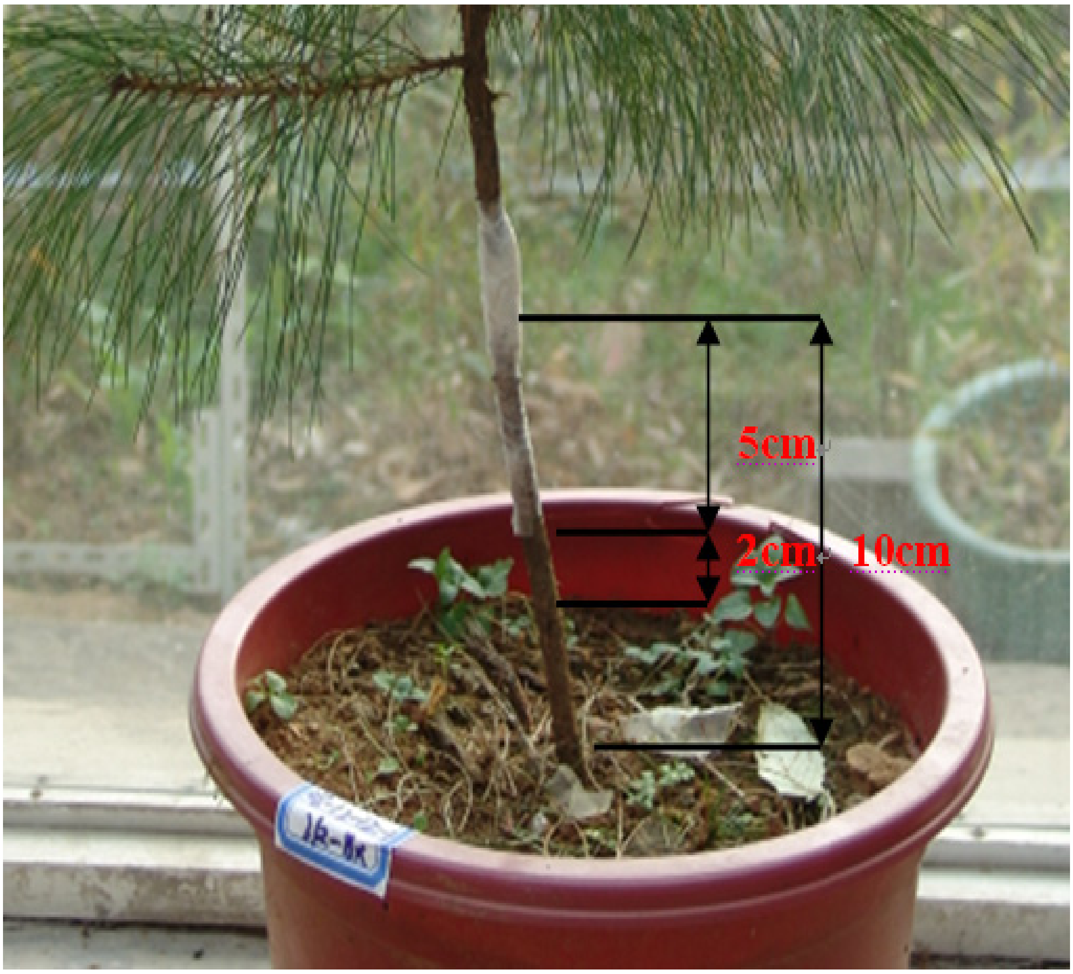

© 2013 by the authors; licensee MDPI, Basel, Switzerland. This article is an open access article distributed under the terms and conditions of the Creative Commons Attribution license (http://creativecommons.org/licenses/by/3.0/).
